# *De novo* transcriptome assembly of the oak processionary moth *Thaumetopoea processionea*

**DOI:** 10.1186/s12863-024-01237-7

**Published:** 2024-06-08

**Authors:** Johan Zicola, Prasad Dasari, Katharina Klara Hahn, Katharina Ziese-Kubon, Armin Meurer, Timo Buhl, Stefan Scholten

**Affiliations:** 1 Division of Crop Plant Genetics, Department of Crop Science, Georg-August-University Göttingen, Göttingen, Germany; 2 Department of Dermatology, University Medical Center Göttingen, Göttingen, Germany; 3 Center for integrated Breeding Research (CiBreed), Göttingen, Germany; 4 Faculty of Resource Management, University of Applied Sciences and Arts (HAWK), Göttingen, Germany

**Keywords:** Transcriptome, RNA-seq, Oak processionary moth, Allergen

## Abstract

**Objectives:**

The oak processionary moth (OPM) (*Thaumetopoea processionea*) is a species of moth (order: *Lepidoptera*) native to parts of central Europe. However, in recent years, it has become an invasive species in various countries, particularly in the United Kingdom and the Netherlands. The larvae of the OPM are covered with urticating barbed hairs (setae) causing irritating and allergic reactions at the three last larval stages (L3-L5). The aim of our study was to generate a *de novo* transcriptomic assembly for OPM larvae by including one non-allergenic stage (L2) and two allergenic stages (L4 and L5). A transcriptomic assembly will help identify potential allergenic peptides produced by OPM larvae, providing valuable information for developing novel therapeutic strategies and allergic immunodiagnostic assays.

**Data:**

Transcriptomes of three larval stages of the OPM were *de novo* assembled and annotated using Trinity and Trinotate, respectively. A total of 145,251 transcripts from 99,868 genes were identified. Bench-marking universal single-copy orthologues analysis indicated high completeness of the assembly. About 19,600 genes are differentially expressed between the non-allergenic and allergenic larval stages. The data provided here contribute to the characterization of OPM, which is both an invasive species and a health hazard.

## Objectives

The impact of the OPM on human health is a significant concern [1]. Direct contact with the caterpillars or their setae containing potential allergenic peptides that can cause skin irritation, redness, itching, and the formation of painful rashes and blisters. In addition to dermatitis, the inhalation of the caterpillar hairs can lead to respiratory problems [2, 3]. The microscopic hairs can irritate the airways, causing symptoms such as coughing, wheezing, sore throat, and difficulty breathing [4]. In some cases, severe allergic reactions may occur, leading to asthma attacks or anaphylaxis, a life-threatening condition. To identify OPM allergens, we generated transcriptomic data for OPM larvae at the non-allergenic stage (L2) and at two allergenic stages (L4 and L5). The *de novo* transcriptomic assembly across all three stages defined the expressed genes and the predicted encoded peptides. Differential gene expression between the stages can highlight genes potentially involved in the allergenic properties of stages L4 and L5. These data will help identifying potential allergenic peptides produced by OPM larvae that can prospectively fill the diagnostic gap in the development of allergic immunization assays and allergy immunotherapy options.

## Data description

### RNA isolation and library preparation

Larvae of *Thaumetopoea processionea* were all collected from a single nest in an English oak tree (*Quercus robur*) in Briesener Zootzen (Germany, 52°45’18.6"N 12°40’29.3"E), in May 14, 2022 (L2 and L4 stages) and June 15, 2022 (L5 stage). The larvae were then brought to the laboratory, snap frozen in liquid nitrogen, and stored at -80 °C. Larvae were homogenized with mortar and pestle in liquid nitrogen and 20 mg of tissue was used for total RNA extraction with the Quick-RNA™ Tissue/Insect Microprep kit (Zymo, R2030). Eleven RNA-seq libraries (4 x L2 larvae, 4 x L4 larvae, 3 x L5 larvae) were prepared with NEBNext® Ultra™ II Directional RNA Library Prep Kit for Illumina® (NEB, E7760L). Paired-end sequencing (100 + 100 bp) was performed on the 11 pooled libraries on the MGISEQ-2000 (BGI) to obtain about 30–55 million reads per library.

### Data filtering, transcriptome assembly and quality

We used the *de novo* transcriptome assembly pipeline recommended by the Harvard Faculty of Arts and Sciences Informatics Group (https://github.com/harvardinformatics/TranscriptomeAssemblyTools) which considers common issues [5]. The raw reads were first cleaned from rare kmers and sequencing errors using Rcorrector [6]. The read adaptors were then trimmed and bad quality reads were removed using cutadapt [7] (cutadapt -a AGATCGGAAGAGCACACGTCTGAACTCCAGTCA -A AGATCGGAAGAGCGTCGTGTAGGGAAAGAGTGT --quality-base 33 --max-n 0 -o output.R1.fq -p output.R2.fq input.R1.fq input.R2.fq). Ribosomal RNA sequences were removed using bowtie2 [8] against the *Lepidoptera* SSU and LSU rRNA sequences downloaded from the SILVA database (https://www.arb-silva.de) (bowtie2 --nofw --quiet --very-sensitive-local --phred33 -x index_bowtie − 1 input.R1.fq -2 input.R2.fq --un-conc-gz output.rRNA_removed.fq.gz > /dev/null). Over-represented sequences were removed using the python script RemoveFastqcOverrepSequenceReads.py (https://github.com/harvardinformatics/TranscriptomeAssemblyTools). Empty reads produced by cutadapt (header present but read sequence removed) were removed using a perl command (perl -i -p -e ‘s/^$/N/g;’ input.fq). The *de novo* assembly of the OPM transcriptome was performed using Trinity (v2.15.1) [9] using the pooled fastq files to build all possible transcripts across all three stages and biological replicates (Trinity --seqType fq --CPU 8 --max_memory 100G --left pooled.R1.fa --right pooled.R2.fa --SS_lib_type RF --output trinity_output). The assembly fasta file was uploaded on NCBI as transcriptomic shotgun assembly for verification, and transcripts identified as duplicates or matching other kingdoms were removed and resubmitted. Raw fastq files and transcriptome assembly are available in NCBI (**Data file 1).** The description statistics of the assembly generated with the Trinity perl script TrinityStats.pl is available in **Data file 2.**

Long open reading frames and derived peptide sequences were obtained using the Perl scripts TransDecoder.LongOrfs and TransDecoder.Predict, respectively (Haas, BJ. https://github.com/TransDecoder (v5.7.0)).

The completeness of the transcriptome assembly was determined with Benchmarking Universal Single-Copy Orthologs (BUSCO) software (v5.4.3) [10]. Longest isoforms of each gene (99,868 genes total) were retrieved using the get_longest_isoform_seq_per_trinity_gene.pl utility script from Trinity. These isoforms were compared to the 5,286 marker genes from the *Lepidoptera* lineage and the completeness found was 89.3%, including 84.9% and 4.4% of single-copy and duplicated genes, respectively (BUSCO analysis summary in **Data file 3**).

### Annotation

Functional annotation of the transcriptome assembly generated by Trinity was performed with Trinotate (v3.2.2) [11] and provided in **Data file 4**.

### Differential expression analysis

To identify differentially expressed between stages, a salmon (v0.10.2) [12] index was first build on the Trinity output fasta file (salmon index -Trinity.fasta -i Trinity.fasta.salmon.idx), the utility Trinity perl script was then used to perform alignment and abundance estimation on single samples (align_and_estimate_abundance.pl --transcripts Trinity.fasta --gene_trans_map Trinity.fasta.gene_trans_map --samples_file samples.txt --est_methold salmon --SS_lib_type RF). The output salmon quant.sf files from salmon were then imported in R using the tximport and DESeq2 (v1.28.1) packages [13, 14]. Differential expressed genes between stages and between the allergenic and non-allergenic stages were identified. Log fold change shrinkage was performed using the apelgm R package [15]. The lists of differentially expressed genes with an adjusted p-value below 5% for each comparison were summarized in an Excel spreadsheet (**Data File 5**).

**Table 1:** Overview of data files

	Name of data file/data set	File types (file extension)	Data repository and identifier (DOI or accession number)
*Data file 1*	*Sequencing data and transcriptome assembly of Thaumetopoea processionea larval stages*	SRA and TSA files (.fastq, .fasta)	NCBI SRA SRP490249 https://identifiers.org/bioproject: PRJNA1072613 [16]
*Data file 2*	*GenBank assembly record of Thaumetopoea processionea larval stages*	GenBank	GenBank GKRZ00000000.1 https://www.ncbi.nlm.nih.gov/nuccore/GKRZ00000000.1 [17]
*Data file 3*	*Summary statistics of the transcriptome assembly*	Text file (.txt)	Figshare, 10.6084/m9.figshare.25333600.v1 [18]
*Data file 4*	*Benchmarking Universal Single-Copy Orthologues (BUSCO) analysis of the transcriptome assembly*	Text file (.txt)	Figshare, 10.6084/m9.figshare.25333603.v1 [19]
*Data file 5*	*Trinotate annotation report*	Compressed text file (.tsv.gz)	Figshare, 10.6084/m9.figshare.25333753.v1 [20]
*Data file 6*	*Genes differentially expressed between stages and between allergenic and non-allergenic stages*	Excel file (.xls)	Figshare, 10.6084/m9.figshare.25333777.v1 [21]
*Data file 7*	*Bioinformatics script for the de novo transcriptome assembly analysis*	Word document (.docx)	Figshare, 10.6084/m9.figshare.25334269.v1 [22]

## Limitations

The *de novo* transcriptomic analysis of the OPM provided here considered only larval stages of the insect. Thus, the transcripts defined here represent only a fraction of the transcriptome. For instance, genes expressed specifically in the imago cannot be detected with our approach. A more comprehensive picture of the OPM transcriptome would require integrating samples from more developmental stages, e.g. egg, pupa, and imago life stages in a *de novo* transcriptome assembly.

## Data Availability

The raw RNA-seq and the transcriptome assembly are available on the NCBI accession number PRJNA1072613 [16]. See Table 1 and references [17–20] for Figshare results. Detailed bioinformatics scripts are available as a PDF document in Data File 6 [21] and on GitHub (https://github.com/johanzi/OPM_transcriptome_assembly).

## Abbreviations

BUSCO: Bench-marking universal single-copy orthologs
OPM: Oak processionary moth
TSA: Transcriptome shotgun assembly
SRA: Short read archive
